# Supplementary material for: Influenza A Virus Infection Reactivates Human Endogenous Retroviruses Associated with Modulation of Antiviral Immunity
Source: Viruses. 2022 Jul 21;14(7):1591. doi: 10.3390/v14071591 (PMC9320126; doi:10.3390/v14071591)
Supplement: Supplementary file 1 [file viruses-14-01591-s001.zip › MotifAnalysis.zip!/MotifOutput_SC35MdNS1/homerResults/motif2.similar.html]

motif2

## Information for motif2

A
G
T
C
C
G
T
A
G
T
A
C
A
G
T
C
G
T
C
A
C
T
G
A
A
G
C
T
A
T
G
C
C
T
G
A
C
T
A
G
A
G
T
C
C
T
G
A
  
Reverse Opposite:  

A
G
C
T
C
T
A
G
A
G
T
C
G
A
C
T
A
T
C
G
C
T
G
A
A
G
C
T
A
C
G
T
C
T
A
G
A
C
T
G
G
A
C
T
T
C
A
G
  

|  |  |
| --- | --- |
| p-value: | 1e-71 |
| log p-value: | -1.651e+02 |
| Information Content per bp: | 1.788 |
| Number of Target Sequences with motif | 41.0 |
| Percentage of Target Sequences with motif | 53.25% |
| Number of Background Sequences with motif | 234.5 |
| Percentage of Background Sequences with motif | 0.52% |
| Average Position of motif in Targets | 93.8 +/- 56.0bp |
| Average Position of motif in Background | 94.3 +/- 97.0bp |
| Strand Bias (log2 ratio + to - strand density) | 10.0 |
| Multiplicity (# of sites on avg that occur together) | 1.28 |
| Motif File: | file (matrix) reverse opposite |

### Similar de novo motifs found

|  |  |  |  |  |  |  |  |
| --- | --- | --- | --- | --- | --- | --- | --- |
| Rank | Match Score | Redundant Motif | P-value | log P-value | % of Targets | % of Background | Motif file |
| 1 | 0.830 | G T A C G A T C G T C A C T G A A C G T A G T C C G T A A C T G A G T C G T C A | 1e-71 | -165.015614 | 54.55% | 0.59% | motif file (matrix) |
| 2 | 0.877 | C G T A A G T C A G T C C G T A C G T A A C G T A G T C C T G A C T A G A G T C | 1e-70 | -162.763544 | 51.95% | 0.48% | motif file (matrix) |
| 3 | 0.889 | C G T A A G T C A G T C C G T A C G T A A C G T A G T C C T A G C T A G A G T C C G T A A G T C | 1e-69 | -159.541606 | 49.35% | 0.40% | motif file (matrix) |
| 4 | 0.717 | C T G A A G C T C T A G A G T C C G T A G T A C A G T C C G T A C G T A A C G T | 1e-68 | -157.023106 | 50.65% | 0.49% | motif file (matrix) |
| 5 | 0.647 | C G T A C G T A A T G C C T A G A T C G C G T A G T A C G T A C C G T A C T G A A C G T A G T C | 1e-67 | -156.284294 | 49.35% | 0.43% | motif file (matrix) |
| 6 | 0.833 | A G T C C G T A A G T C A G T C C G T A C G T A A C G T A G T C | 1e-61 | -141.435283 | 51.95% | 0.82% | motif file (matrix) |
| 7 | 0.719 | G A T C T C A G C T G A A C G T A C G T A C T G C T A G A G C T | 1e-42 | -97.757964 | 57.14% | 3.56% | motif file (matrix) |
| 8 | 0.622 | A G T C T G C A A G T C G T A C C G T A A C G T G A C T G T A C A C T G C T G A | 1e-14 | -34.485625 | 9.09% | 0.04% | motif file (matrix) |
| 9 | 0.730 | A G T C C G T A A G T C A T G C C G T A A C T G A C G T A G T C | 1e-8 | -18.501570 | 14.29% | 1.37% | motif file (matrix) |
